# Clinical Health Psychology Perspectives in Diabetes Care: A Retrospective Cohort Study Examining the Role of Depression in Adherence to Visits and Examinations in Type 2 Diabetes Management

**DOI:** 10.3390/healthcare12191942

**Published:** 2024-09-27

**Authors:** Rossella Messina, Jacopo Lenzi, Simona Rosa, Maria Pia Fantini, Paolo Di Bartolo

**Affiliations:** 1 Department of Biomedical and Neuromotor Sciences, Alma Mater Studiorum, University of Bologna, 40126 Bologna, Italy; 2 Diabetes Unit, Local Healthcare Authority of Romagna, 48100 Ravenna, Italy

**Keywords:** T2DM, depression, adherence, Guideline Composite Indicator, integrated care

## Abstract

Background: Depression in type 2 diabetes mellitus (T2DM) impacts glycemic control and complications. This study examines the influence of depression on compliance with recommended annual diabetes assessments in patients within the Local Healthcare Authority of Romagna. From a clinical health psychology perspective, understanding how depression influences patients’ engagement in managing their conditions is crucial. This insight can help improve healthcare services by ensuring they address mental health needs and thereby enhance treatment effectiveness and overall patient outcomes. Methods: This retrospective cohort study included residents of Romagna with incident T2DM from 2015 to 2017, followed from 1 January 2018 to 31 December 2022. Depression was identified via hospital discharge records or antidepressant prescriptions. Adherence to diabetes care guidelines was measured using the Guideline Composite Indicator (GCI). Results: The study included 13,285 patients, with a mean age of 61.1 years. Prevalence of post-diabetes depression increased from 3.0% in 2018 to 8.9% in 2022. Initial analyses showed higher GCI rates among patients with depression. However, propensity-score adjustment revealed that by 2021–2022, patients with pre-diabetes depression had 5% lower compliance rates (*p*-value ≤ 0.05). Older adults with depression had reduced adherence, while younger adults with post-diabetes depression had higher adherence rates. Conclusions: Depression significantly affects adherence to diabetes care guidelines in T2DM patients, particularly among older adults. Integrated care models addressing both diabetes and depression are crucial for improving health outcomes.

## 1. Introduction

Individuals with type 2 diabetes mellitus (T2DM) are at a significantly higher risk of developing depression compared to the general population, with evidence suggesting they are twice as likely to experience this comorbidity [1,2,3]. This association exacerbates the severity and outcomes of both conditions, resulting in a bidirectional relationship where each adversely affects the other [4]. Indeed, numerous studies have highlighted the considerable impact of depression on the risk of acute and long-term complications and increased mortality rates in T2DM patients [5,6,7].

Research indicates that depression complicates diabetes management by impairing adherence to essential self-care regimens, such as blood glucose monitoring, diet, physical activity, and medication compliance [8,9]. For example, Lin et al. [10] found a significant relationship between depression and reduced adherence to preventive care in T2DM patients, while Lunghi et al. [11] demonstrated that depression is linked to medication non-persistence in new users of antidiabetic drugs.

Further studies have underscored the complex relationship between depression, medication adherence, and quality of life in T2DM patients. For example, Yang et al. [7] showed that poor medication adherence influenced by depressive symptoms significantly reduces the quality of life for these patients. In this context, health psychology theories such as the Health Belief Model (HBM) and Self-Determination Theory (SDT) offer valuable insights into supporting individuals with depression. The HBM posits that beliefs about health, perceived barriers, and self-efficacy influence health behaviors [12,13], and depression can distort these beliefs, leading to reduced adherence. The SDT emphasizes the importance of autonomy, competence, and relatedness in motivating health behaviors [14], which can be particularly challenging for those with depression. In fact, as suggested by Devarajooh and Chinna [15], depression and distress significantly impact self-care through reduced self-efficacy, which is a psychological construct involved in diabetes management [16,17].

Given the growing recognition of depression as a major complication of T2DM, it is no surprise that the American Diabetes Association’s latest edition of “Standards of Medical Care in Diabetes” recommends annual screenings for depressive symptoms in all people with diabetes, and more frequent screenings for those with a self-reported history of depression [18]. These screenings are crucial for identifying at-risk patients and ensuring timely and integrated interventions, addressing depressive symptoms linked to lower adherence to diabetes care recommendations and suboptimal glycemic control [8,9]. Notably, a recent longitudinal study by Gillett et al. [1] using UK Biobank primary care records found that individuals diagnosed with major depressive disorder (MDD) after their T2DM diagnosis had poorer glycemic control and increased within-patient variability in HbA1c levels. This study highlights the importance of the timing of MDD diagnosis in relation to T2DM, as post-T2DM MDD was associated with a significant increase in HbA1c over time. These findings underscore the need for ongoing depression screening and closer monitoring in T2DM management.

Despite these insights, there is limited research on adherence to annual diabetes care visits and examinations, which are essential for effective management and prevention of complications. This gap underscores the need for further investigation into how depression impacts adherence to these critical healthcare visits. This study aims to investigate adherence to diabetes care guidelines among individuals with T2DM within the Local Healthcare Authority (LHA) of Romagna, with a specific focus on those with comorbid depression.

## 2. Materials and Methods

### 2.1. Setting and Data Sources

This retrospective cohort study followed the STROBE reporting guidelines (see Supplementary Table S1) and included all residents in the LHA of Romagna with an estimated onset of T2DM between 2015 and 2017. The follow-up period spanned from 1 January 2018 to 31 December 2022 (see Section 2.3 for details). Romagna’s LHA, located in Northeastern Italy, served approximately 1,125,000 inhabitants as of 1 January 2024.

Data sources, each with an anonymized patient identifier, included the following healthcare administrative datasets (see Supplementary Table S2 for details): (I) Hospital Discharge Records; (II) Residential Care Discharge Records; (III) Mental Health Information System; (IV) Outpatient Pharmaceutical Database and Direct Pharmaceutical Delivery Database; (V) Specialist Ambulatory Care; (VI) Vital Registration System.

### 2.2. Inclusion and Exclusion Criteria

Incident cases of T2DM were identified based on meeting one of the following inclusion criteria [6,19]:

- Hospital admission with a primary or secondary diagnosis of diabetes (coded as 250 under the International Classification of Diseases, Ninth Revision, Clinical Modification [ICD-9-CM]) and at least one filled prescription for glucose-lowering medication (coded as A10 under the Anatomical Therapeutic Chemical [ATC] Classification System);
- Three separate prescriptions for glucose-lowering medications.

Uncertain cases of T2DM, such as those where insulin was the initial and only treatment in the first year, or women with gestational diabetes (ICD-9-CM code 648.8) were not considered for inclusion in the study. Additional exclusion criteria included the following: (I) individuals under 25 years of age; (II) current or prior diabetes complications occurring within three years before the estimated onset of diabetes (see Supplementary Table S3 for details) [6,19]. After applying these criteria, 13,756 eligible patients remained from the initial 15,946 (86.3%). Excluded patients are partitioned as follows: (I) 188 younger than 25; (II) 2002 with current or prior diabetes complications.

### 2.3. Process Indicators for Quality of Diabetes Care

According to the evidence-based international, national and regional recommendations [20,21,22], receipt and quality of care were measured as having an annual assessment of the following: (I) glycated hemoglobin (HbA1c) for glycemic control; (II) total cholesterol, high-density lipoprotein (HDL) cholesterol, or triglycerides for lipid profile; (III) microalbuminuria, creatinine clearance (i.e., glomerular filtration), or creatinine for renal function; (IV) eye screening, including examination of the fundus oculi, picture of the fundus oculi, or a comprehensive eye examination. These visits and examinations were extracted from the Specialist Ambulatory Care administrative dataset. We used the Guideline Composite Indicator (GCI) proposed by Profili et al. [23] for the region of Tuscany as an overall measure of compliance with follow-up standards of care management. The GCI was imputed as one (“adherent”) if the patient had an annual assessment of HbA1c and at least two annual assessments among lipid profile, renal function, and eye screening.

The GCI was computed each year between 2018 and 2022, for a total of five annual evaluations. Specifically, patients included in each year-specific analysis were those alive on January 1, and contributed to the annual denominator with one, or less in case of death occurring before December 31. Starting the observation period in 2018 means that the first set of indicators was calculated for patients with an estimated onset of T2DM up to a maximum of three years earlier. Similarly, ending the observation period in 2022 implies that the last set of indicators was provided for patients with a history of diabetes dating back four to seven years.

Out of the initial study population of 13,756, the number of patients included in the annual cohorts for 2018–2022 were 13,285 (96.6%), 13,025 (94.7%), 12,770 (92.8%), 12,461 (90.6%), and 12,111 (88.0%), respectively. Overall, the mean follow-up time was 4.7 ± 0.9 years, with a total of 62,888.8 patient-years analyzed.

### 2.4. Exposure to Depression

Depression was identified based on an inpatient or outpatient visit with a diagnosis of depression (ICD-9-CM) or through a filled prescription for antidepressant medications (ATC code N06A) [6,19]. Additional details can be found in Supplementary Table S3.

Two mutually exclusive exposure variables were considered for longitudinal data analysis [1]: (I) past depression within three years before the estimated onset of diabetes; (II) development of depression during follow-up, specifically after the estimated onset of diabetes up to January 1st of each year between 2018 and 2022. This resulted in a single exposure variable with three categories: no depression, pre-diabetes depression, and post-diabetes depression.

In a secondary analysis limited to patients with post-diabetes depression, we assessed the impact of disease duration, defined as the number of years from depression onset to January 1st of each year, on adherence to guidelines as expressed by the GCI. Consequently, depression duration varied by construction according to the year being analyzed: up to three years for 2018, up to four years for 2019, up to five years for 2020, up to six years for 2021, and up to seven years for 2022.

### 2.5. Potential Confounders

Individual characteristics considered for potential confounding in propensity-score analysis were the following:

- Sex (male or female);
- Age at diabetes onset (in years);
- Citizenship (Italian or non-Italian);
- Year of onset (2015, 2016 or 2017);
- Drugs within 30 days of onset (one oral antidiabetic, two or more oral antidiabetics, insulin only, or oral antidiabetics plus insulin), as a proxy for timeliness of diagnosis and glycemic control at disease onset;
- Thirty-one clinical conditions retrieved up to three years prior to onset and summarized using the Multisource Comorbidity Score (MCS), which is calculated by adding the specific weights assigned to each condition (diabetes and acute myocardial infarction were not included in the MCS calculation because our study population, by definition, did not have any of these conditions before their entry into the study) [24];
- Health district of residence (a total of eight).

In Italy, health districts oversee the provision of primary care and coordinate local requests for specialist services, diagnostics, and hospital care, both for outpatients and inpatients. Although LHAs aim to deliver high-quality services consistently, there may be differences in organizational models across health districts.

### 2.6. Statistical Analysis

Categorical variables were reported as counts and percentages, while numerical variables were expressed as mean ± standard deviation.

For each year of follow-up (2018–2022), crude comparisons of adherence to guideline recommendations (GCI) across depression groups were conducted using Poisson regression analysis with a robust error variance procedure, also known as sandwich estimation [25]. This approach accounts for deviations from the assumptions of homoskedasticity, independence, and equidispersion. Different observation periods within the year for each patient were handled as an offset variable.

The analysis was replicated with the aid of multivariable Poisson regression analysis adjusted for potential confounding through a priori covariates and weights based on propensity-for-depression scores. Firstly, Multiple Additive Regression Trees (MART) gradient boosting was employed to estimate the propensity for depression using the explanatory variables listed in Section 2.5 [26,27]. Regularization settings included the following: maximum tree depth of five interactions; maximum of 20,000 iterations; 50% bagging; 0.01 shrinkage factor. Given the multivalued outcome (no depression, pre-diabetes depression, and post-diabetes depression), a multinomial distribution was used to obtain depression predictions from the boosting models. Secondly, because the estimand of interest was the average effect of depression in the population suffering from this condition (analogous to the average treatment effect on the treated [ATET]), each observation was weighted by the reciprocal of the probability of its actual depression status, multiplied by the probability of being depressed and distinguishing between pre- and post-diabetes depression diagnoses. Year- and exposure-specific weights were truncated at the 90th percentile, which means that weights larger than the 90th percentile were replaced with the value of the 90th percentile. This approach aimed to remedy extreme weights, mitigate bias and variance of effect estimates, and ensure sufficient propensity-score overlap between groups while preserving statistical power [28,29,30]. Thirdly, a doubly robust estimation strategy was applied to account for possible residual confounding after boosting modeling [28,29]. All covariates were centered at the unweighted mean and included as covariates in the regression Poisson models with robust variance, allowing for squared terms to enhance model specification. Lastly, analyses were stratified by age group (< 65 vs. ≥ 65 years) to evaluate whether the effect of depression on compliance with diabetes health checks differed between adults and older adults.

A secondary analysis on disease duration for subjects with post-diabetes depression (see Section 2.4) was conducted using covariate adjustment.

Effect sizes derived from Poisson modeling were expressed as incidence rate ratios (IRRs). All analyses were conducted with Stata 18 (StataCorp, 2023, Stata Statistical Software: Release 18. College Station, TX, USA: StataCorp LLC). The significance level was set at 0.05, and all tests were two-tailed.

### 2.7. Ethics Statement

Approval for this research was granted by the Ethics Committee of Romagna’s LHA on 14 December 2020 (Registration #9502/2020), with reapproval for extension on 27 September 2023 (Registration #5869/2023).

Emilia-Romagna’s health administrative data undergo pseudonymization at the regional statistical office prior to analysis. Each patient is assigned a unique identifier, ensuring that identities cannot be traced or accessed for other sensitive data. In accordance with Article 9 of the General Data Protection Regulation (European Union (EU) Regulation 2016/679), pseudonymized administrative data can be used for healthcare management, quality evaluation, and improvement without requiring specific written informed consent from patients. The study adhered to the principles of the 1964 Helsinki Declaration and its subsequent amendments.

## 3. Results

The characteristics of the 13,285 patients enrolled in the cohort as of 1 January 2018 are summarized in Table 1. The mean age at T2DM onset was 61.1 ± 15.0 years, with females comprising 51.0% of the population. A majority (86.1%) held Italian citizenship, 80.3% had an MCS ≤ 4, and 85.7% were initially prescribed a single oral antidiabetic medication. Patients with depression, developed either before or after diabetes onset, were more frequently female, older, Italian citizens, and had higher MCSs compared to patients without depression.

As shown in Table 2, the prevalence of depression following diabetes onset increased from 3.0% in 2018 to 8.9% in 2022. In contrast, due to the study design and the reduction in the population size due to mortality, the prevalence of depression prior to diabetes onset slightly decreased from 15.8% to 14.7%.

As displayed in Table 3, the GCI for recommended good management in 2018 was 53.0 per 100 person-years. According to IRRs, GCI rates were significantly higher among patients with pre- and post-diabetes depression. Compliance declined in 2020, with a GCI of 48.3 per 100 person-years, yet both depression groups maintained higher IRRs for received examinations. By 2022, GCI rates had returned to levels similar to those observed at the beginning of the observation period.

**Figure 1:**
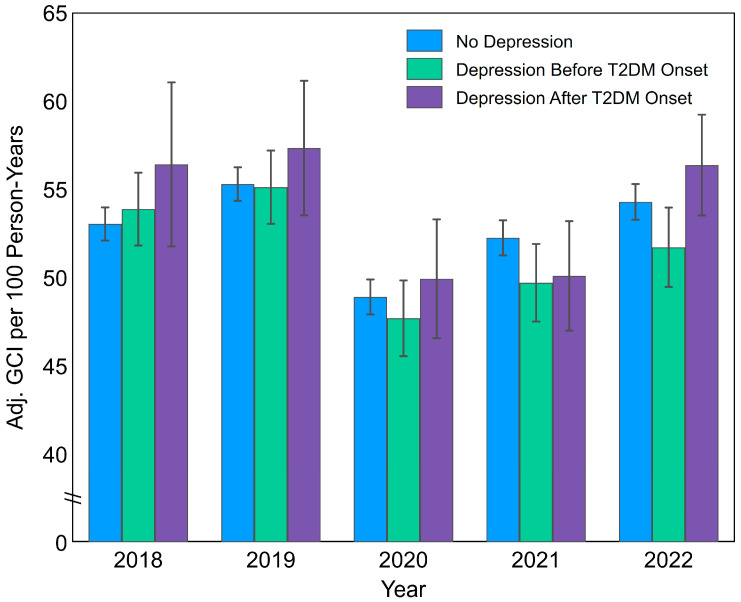
Results of propensity-score analysis using inverse probability weighting and covariate adjustment: rates of recommended annual exams (GCI per 100 person-years) among patients with onset of T2DM in 2015–2017, followed up in 2018–2022, overall and by depression status. Notes: Depression before diabetes was tracked up to three years before diabetes onset. Point estimates of GCI rates are presented along with 95% confidence intervals. Abbreviations: GCI, Guideline Composite Indicator.

After accounting for confounding via propensity-score analysis (Figure 1 and Table 4), neither depression group exhibited significantly improved GCI rates. On the contrary, patients with evidence of pre-diabetes depression had lower compliance in 2021–2022, with an adjusted IRR of 0.95 (*p*-value ≤ 0.05) for both years.

Compliance with recommended exams for diabetes care was generally higher among patients aged ≥65 years at disease onset (Table 5). However, after accounting for confounding, adherence to guidelines in this subpopulation was significantly lower for both depression groups over the years 2021–2022. In contrast, among patients aged <65 years, we found significantly higher adherence among those with post-diabetes depression compared to those with no evidence of depression.

The results of the secondary analysis on depression duration are summarized in Supplementary Table S4. While a relative reduction in GCI was observed with one-year increases in post-diabetes depression, none of the IRRs were statistically significant.

## 4. Discussion

This retrospective cohort study examined the impact of pre-diabetes and post-diabetes depression on adherence to recommended annual visits and examinations over five years (2018–2022) within the LHA of Romagna. The main findings underscore that depression, whether diagnosed prior to or following the onset of diabetes, significantly impacts compliance with recommended annual assessments, although the degree and direction of this association vary.

Consistent with the existing literature, our results confirm that depression is a prevalent comorbidity in patients with T2DM, with significant implications for diabetes management. Previous studies have documented that depression adversely affects self-care behaviors, including adherence to medication, dietary recommendations, and physical activity—all critical components of diabetes management [31,32]. This study expands on these findings by specifically examining adherence to annual diabetes care assessments as measured by the GCI [23].

Our initial analysis revealed that patients with depression, both before and after diabetes onset, exhibited higher GCI rates compared to those without depression. This finding, which may seem counterintuitive, may reflect increased healthcare engagement or more frequent monitoring due to the perceived health risks associated with comorbid depression. However, propensity-score analysis indicated that this association was likely spurious, primarily due to the confounding effect of older age, which was associated with both depression and higher adherence to guidelines. Alongside a loss of statistical significance for the period 2018–2020, we also observed that by 2021–2022, patients with pre-diabetes depression had significantly lower compliance rates (IRR = 0.95, *p*-value ≤ 0.05). This decline may be indicative of the broader repercussions of the COVID-19 pandemic on mental health and healthcare engagement. The pandemic disrupted daily routines and access to healthcare services [33]. Social distancing measures and lockdowns likely reduced access to in-person care, while increased stress and social isolation may have worsened depressive symptoms, negatively affecting adherence to treatment plans [34,35]. Additionally, the strain on healthcare systems and the shifting priorities during the pandemic could have compromised the continuity and quality of care for individuals with chronic conditions.

The age-stratified analysis revealed distinct patterns in adherence. Among patients aged 65 years and older, those with depression (both pre- and post-diabetes) exhibited lower adherence to guidelines in the later years of follow-up. This suggests that older adults with depression face additional barriers to maintaining consistent diabetes care, potentially due to greater disease burden, cognitive decline, or reduced social support [1]. Moreover, disruption in routine healthcare, combined with the psychological toll of isolation and stress, may have significantly contributed to decreased adherence. Conversely, among younger adults (<65 years), post-diabetes depression was associated with higher adherence rates compared to their non-depressed counterparts. This may indicate that younger individuals with depression receive more targeted interventions or have different coping mechanisms and support systems that facilitate adherence to care [36], despite the challenging circumstances posed by the COVID-19 pandemic. This discrepancy in adherence rates between age groups underscores the importance of age-specific interventions and support systems to address the distinct needs of populations during health crises. Future pandemics and public health emergencies should prioritize maintaining continuity of care for vulnerable populations, such as older adults with comorbid chronic conditions and mental health disorders. Strategies may include expanding telehealth services, enhancing social support networks, and ensuring that healthcare systems remain adaptable to the unique needs of these populations. These findings highlight the need for proactive, age-tailored interventions to mitigate the long-term effects of pandemics on healthcare adherence and chronic disease management.

Adherence rates to diabetes care guidelines were low across the entire study population. The GCI rates hovered at around 50% throughout the study period, indicating that only about half of the patients consistently received the recommended annual exams. This low adherence rate is concerning because comprehensive diabetes management, including regular monitoring of glycemic control, lipid profiles, renal function, and eye health, is crucial for preventing complications and optimizing health outcomes. These findings highlight a critical need for interventions aimed at improving adherence to diabetes care guidelines across the board. Potential strategies could include enhanced patient education, better access to healthcare services, and more robust support systems, particularly for those with additional risk factors such as depression.

Supporting patients’ psychological needs through integrated care can enhance motivation and adherence [37]. The recent theory of Psychological Adjustment to Long-Term Conditions (TMA-LTC) highlights how acute events or ongoing stressors disrupt emotional equilibrium. Psychological adjustment depends on cognitive and behavioral factors, emphasizing the need to address illness-specific challenges and promote adaptive coping [38]. Recently, a randomized control trial [39] evaluating the efficacy of Cognitive Behavioral Therapy (CBT) to treat depressive symptoms in individuals with T2DM demonstrated significant reductions in depressive symptoms and anxiety, alongside improvements in quality of life, treatment adherence, and physical activity. Shah et al. [40] and Sasseville et al. [41] further underscore the importance of Digital Health Interventions (DHIs) in supporting both mental health and chronic disease management. Shah et al. [40] emphasize that DHIs tailored to individuals with chronic conditions, including T2DM, effectively reduce depressive symptoms and improve treatment adherence. Similarly, Sasseville et al. [41] show that digital CBT interventions lead to significant improvements in both mental health outcomes and diabetes management. These findings highlight the potential of technology-driven interventions to address the dual challenge of depression and T2DM, particularly in enhancing patient motivation, self-management, and healthcare engagement. Moreover, the collaborative care approach has been shown to be effective in reducing depression and improving quality of life in people with comorbid depression and diabetes [42].

### Strengths and Limitations

The study has significant strengths, particularly the large cohort size of 13,285, which provides considerable statistical power and enhances the external validity of the findings. The use of propensity-score analysis to adjust for potential confounders further strengthens the validity of the findings by minimizing bias.

There are also important limitations that must be acknowledged. The most significant is the “static” nature of the cohort, which consists of patients with an estimated onset of T2DM between 2015 and 2017, without subsequent updates. Ideally, a “dynamic” cohort would have been preferable, allowing the inclusion of new diabetes cases on a yearly basis. Such an approach would have enabled us to better disentangle the effects of the pandemic and the duration of T2DM on both depression exposure and adherence to guidelines. As a result, the explanation for the significantly different GCI rates observed according to depression status during 2021–2022 remains challenging. These differences could be attributed to the pandemic’s impact or to broader difficulties in maintaining consistent diabetes care over time.

However, further exploration of the GCI rate trends throughout the study period provides additional context. As shown in Table 3 and Figure 1, GCI rates exhibited a marked decline in 2020 followed by a return to pre-COVID-19 levels by 2022. This recovery in 2022 suggests a potential normalization of diabetes care practices as healthcare systems adapted to the challenges posed by the pandemic. It also highlights the possibility that patients with depression may have experienced more pronounced disruptions in care during the pandemic, which could account for the observed differences in adherence. While this remains partly speculative, the trend in GCI rates supports the notion that the pandemic had a temporary but significant impact on diabetes management, disproportionately affecting certain subgroups, such as those with co-occurring depression. Linear or stable trends, in contrast, would have supported alternate interpretations based on disease duration and aging.

In summary, although we acknowledge this important limitation, we believe that our findings remain valuable in understanding the impact of mental health on diabetes management during a period of substantial healthcare disruption. Future studies based on dynamic cohorts may build upon these initial insights and provide a clearer picture of how the pandemic has influenced chronic disease management in patients with T2DM and depression.

Other limitations include the use of the GCI, an adherence metric that does not capture all relevant aspects of patient behavior, such as daily self-monitoring of blood glucose, medication use, and dietary adherence. Another limitation is the case definition of depression, which relies on filled prescriptions or hospitalizations. This algorithm likely suffers from low specificity, leading to a high rate of false positives. While it is difficult to ascertain the exact direction of bias introduced by this misclassification, we can reasonably argue that the strength of the association between depression and GCI was likely underestimated. Moreover, reliance on administrative healthcare data may overlook nuanced patient behaviors and clinical decision-making not documented in these records. These databases also lack crucial information such as socioeconomic status, proximity to healthcare facilities, transportation barriers—factors that could have enhanced propensity-score adjustment and strengthened recommendations for arguing more tailored interventions. Lastly, the focus on the LHA of Romagna may have limited the study’s generalizability. In fact, variations in healthcare systems and cultural contexts may affect the transferability of our results to other regions or populations.

Overall, despite these limitations, our findings highlight the need for integrated care models that address both the psychological and physical health needs of patients with T2DM. Depression screening and management should be integral components of diabetes care pathways [18] to help identify individuals at risk for poor adherence and tailor interventions accordingly. In fact, by integrating mental health assessments into diabetes care, healthcare providers can better identify and address co-occurring conditions that may impact diabetes management. Further qualitative research could complement this evidence by providing deeper insights into the barriers and facilitators affecting diabetes self-care among patients experiencing depression.

## 5. Conclusions

Addressing depression in patients with T2DM is crucial for improving adherence to the comprehensive medical evaluations recommended in diabetes care guidelines. Future research should focus on developing and evaluating integrated care models that concurrently address diabetes and depression, particularly among older adults who appear to be at greater risk of nonadherence. Systemic efforts are needed to elevate adherence rates in people with T2DM to ensure comprehensive and effective diabetes management.

## Figures and Tables

**Table 1:** Characteristics of the study population in the Local Healthcare Authority of Romagna with T2DM onset in 2015–2017, overall and by depression status as of 1 January 2018.

	All (*n* = 13,285)	No Depression (*n* = 10,789)	Depression	Depression
			before	after
			Diabetes Onset *	Diabetes Onset
			(*n* = 2095)	(*n* = 401)
Sex				
Male	6512 (49.0%)	5676 (52.6%)	677 (32.3%)	159 (39.7%)
Female	6773 (51.0%)	5113 (47.4%)	1418 (67.7%)	242 (60.3%)
Age, y	61.1 ± 15.0	60.1 ± 14.9	65.6 ± 14.4	65.0 ± 15.6
Age Group, y				
25–34	778 (5.9%)	718 (6.7%)	47 (2.2%)	13 (3.2%)
35–44	1255 (9.4%)	1096 (10.2%)	120 (5.7%)	39 (9.7%)
45–54	2148 (16.2%)	1786 (16.6%)	314 (15.0%)	48 (12.0%)
55–64	3159 (23.8%)	2647 (24.5%)	432 (20.6%)	80 (20.0%)
65–74	3252 (24.5%)	2614 (24.2%)	543 (25.9%)	95 (23.7%)
75–84	2130 (16.0%)	1574 (14.6%)	463 (22.1%)	93 (23.2%)
≥85	563 (4.2%)	354 (3.3%)	176 (8.4%)	33 (8.2%)
Citizenship				
Italian	11,440 (86.1%)	9106 (84.4%)	1980 (94.5%)	354 (88.3%)
Non-Italian	1845 (13.9%)	1683 (15.6%)	115 (5.5%)	47 (11.7%)
Health District of Residence				
Ravenna	2745 (20.7%)	2266 (21.0%)	414 (19.8%)	65 (16.2%)
Lugo	1456 (11.0%)	1139 (10.6%)	267 (12.7%)	50 (12.5%)
Faenza	1117 (8.4%)	899 (8.3%)	189 (9.0%)	29 (7.2%)
Forlì	1930 (14.5%)	1552 (14.4%)	315 (15.0%)	63 (15.7%)
Cesena—Valle del Savio	1389 (10.5%)	1120 (10.4%)	226 (10.8%)	43 (10.7%)
Rimini	2367 (17.8%)	1924 (17.8%)	361 (17.2%)	82 (20.4%)
Riccione	1216 (9.2%)	1012 (9.4%)	167 (8.0%)	37 (9.2%)
Rubicone	1065 (8.0%)	877 (8.1%)	156 (7.4%)	32 (8.0%)
Year of Diabetes Onset				
2015	4272 (32.2%)	3425 (31.7%)	654 (31.2%)	193 (48.1%)
2016	4302 (32.4%)	3443 (31.9%)	714 (34.1%)	145 (36.2%)
2017	4711 (35.5%)	3921 (36.3%)	727 (34.7%)	63 (15.7%)
MCS †	2.8 ± 4.7	2.3 ± 4.3	5.1 ± 6.0	3.7 ± 5.5
Grouped MCS †				
≤4	10,672 (80.3%)	9132 (84.6%)	1250 (59.7%)	290 (72.3%)
5–9	1685 (12.7%)	1124 (10.4%)	487 (23.2%)	74 (18.5%)
10–14	555 (4.2%)	325 (3.0%)	212 (10.1%)	18 (4.5%)
15–19	201 (1.5%)	104 (1.0%)	88 (4.2%)	9 (2.2%)
≥20	172 (1.3%)	104 (1.0%)	58 (2.8%)	10 (2.5%)
Initial Diabetes Treatment ‡				
One oral medication	11,391 (85.7%)	9226 (85.5%)	1828 (87.3%)	337 (84.0%)
Two or more oral medications	976 (7.3%)	802 (7.4%)	142 (6.8%)	32 (8.0%)
Insulin only	399 (3.0%)	320 (3.0%)	65 (3.1%)	14 (3.5%)
Oral medication(s) and insulin	519 (3.9%)	441 (4.1%)	60 (2.9%)	18 (4.5%)

* Tracked up to three years before diabetes onset. † Diabetes and acute myocardial infarction were not included in the MCS calculation. ‡ Within 30 days of diabetes onset. Abbreviations: LHA, Local Healthcare Authority; IQR, Interquartile Range; MCS, Multisource Comorbidity Score.

**Table 2:** Population size and depression prevalence as of 1 January for the years 2018–2022.

	Year 2018		Year 2019		Year 2020		Year 2021		Year 2022	
	(*n* = 13,285)		(*n* = 13,025)		(*n* = 12,770)		(*n* = 12,461)		(*n* = 12,111)	
	*n*	%	*n*	%	*n*	%	*n*	%	*n*	%
No Depression	10,789	81.2	10,370	79.6	9991	78.2	9646	77.4	9259	76.5
Depression Before Diabetes *	2095	15.8	2027	15.6	1964	15.4	1873	15.0	1777	14.7
Depression After Diabetes	401	3.0	628	4.8	815	6.4	942	7.6	1075	8.9

* Tracked up to three years before diabetes onset.

**Table 3:** Rates of recommended annual exams (GCI per 100 person-years) among patients with onset of T2DM in 2015–2017, followed up in 2018–2022, overall and by depression status.

Year			Depression	Depression		
	All	No	before	after	Incidence Rate Ratios	
	Patients	Depression	Diabetes	Diabetes		
		(1)	Onset * (2)	Onset (3)	(2) vs. (1)	(3) vs. (1)
2018	53.0%	51.8%	58.0%	58.2%	1.12 †	1.12 †
2019	54.8%	53.7%	59.3%	58.4%	1.10 †	1.09 †
2020	48.3%	47.2%	52.5%	51.7%	1.11 †	1.10 †
2021	51.4%	51.0%	53.6%	51.2%	1.05 †	1.01
2022	53.7%	53.0%	55.5%	56.8%	1.05 †	1.07 †

* Tracked up to three years before diabetes onset. † *p*-value ≤ 0.05. Abbreviations: GCI, Guideline Composite Indicator.

**Table 4:** Results of propensity-score analysis using inverse probability weighting and covariate adjustment: incidence rate ratios of recommended annual exams for T2DM (GCI) in patients with pre- and post-diabetes depression compared to those without depression.

	Depression before Diabetes			Depression after Diabetes		
Year	Onset * vs. No Depression			Onset vs. No Depression		
	IRR	95% CI	*p*-Value	IRR	95% CI	*p*-Value
2018	1.02	0.97, 1.06	0.479	1.06	0.98, 1.16	0.153
2019	1.00	0.96, 1.04	0.879	1.04	0.97, 1.11	0.306
2020	0.98	0.93, 1.03	0.325	1.02	0.95, 1.10	0.569
2021	0.95	0.91, 1.00	0.047 †	0.96	0.90, 1.02	0.216
2022	0.95	0.91, 1.00	0.049 †	1.04	0.98, 1.10	0.194

* Tracked up to three years before diabetes onset. † *p*-value ≤ 0.05. Notes: IRRs express the average effect of depression in the population with T2DM suffering from this condition. Abbreviations: GCI, Guideline Composite Indicator; IRR, Incidence Rate Ratio; CI, Confidence Interval.

**Table 5:** Rates of recommended annual exams (GCI per 100 person-years) among patients aged <65 vs. ≥65 years with T2DM onset in 2015–2017, followed up in 2018–2022, overall and by depression status.

	All Patients	No Depression (1)	Depression before	Depression after	Crude IRRs		Adjusted IRRs	
			Diabetes Onset * (2)	Diabetes Onset (3)	(2) vs. (1)	(3) vs. (1)	(2) vs. (1)	(3) vs. (1)
Adults (<65 y)								
2018	46.1%	44.8%	53.4%	52.9%	1.19 †	1.18 †	1.04	1.09
2019	47.9%	46.7%	54.2%	52.9%	1.16 †	1.13 †	1.04	1.08
2020	42.3%	41.1%	47.2%	49.5%	1.15 †	1.20 †	0.99	1.09
2021	46.0%	45.2%	49.8%	50.3%	1.10 †	1.11 †	0.98	1.14 †
2022	48.4%	47.1%	51.5%	57.1%	1.09 †	1.21 †	0.97	1.19 †
Older Adults (≥65 y)								
2018	61.6%	61.6%	61.6%	62.6%	1.00	1.02	1.00	1.03
2019	63.6%	63.7%	63.4%	63.1%	1.00	0.99	0.97	1.01
2020	56.4%	56.5%	57.1%	53.5%	1.01	0.95	0.96	0.95
2021	58.9%	60.2%	57.1%	52.0%	0.95	0.86 †	0.93 †	0.87 †
2022	61.5%	62.8%	59.5%	56.5%	0.95	0.90 †	0.94 †	0.93 †

* Tracked up to three years before diabetes onset. † *p*-value ≤ 0.05. Notes: IRRs obtained via propensity scores and covariate adjustment (“adjusted”) express the average effect of depression in the population with T2DM suffering from this condition. Abbreviations: GCI, Guideline Composite Indicator; IRR, Incidence Rate Ratio.

## Data Availability

The data presented in this study are available on request from the corresponding author due to restrictions imposed by the Ethics Committee’s policy.

## Supplementary Materials

The following supporting information can be downloaded at: https://www.mdpi.com/article/10.3390/healthcare12191942/s1, Table S1: STROBE reporting guidelines; Table S2: Description of data sources; Table S3: ICD-9-CM Diagnosis Codes; Table S4: Adjusted Incidence Rate Ratios of GCI.
