# Supplementary material for: Clinical Health Psychology Perspectives in Diabetes Care: A Retrospective Cohort Study Examining the Role of Depression in Adherence to Visits and Examinations in Type 2 Diabetes Management
Source: Healthcare (Basel). 2024 Sep 27;12(19):1942. doi: 10.3390/healthcare12191942 (PMC11475538; doi:10.3390/healthcare12191942)
Supplement: Supplementary file 1 [file healthcare-12-01942-s001.zip › healthcare-3177249-supplementary.pdf]

SUPPLEMENTARY MATERIAL

Table S1: STROBE reporting guidelines

	Item No.	Recommendation	Page No
**Title and abstract**	1	(*a*) Indicate the study's design with a commonly used term in the title or the abstract	1
		(*b*) Provide in the abstract an informative and balanced summary of what was done and what was found	1
**Introduction**			
Background/rationale	2	Explain the scientific background and rationale for the investigation being reported	1–2
Objectives	3	State specific objectives, including any prespecified hypotheses	2
**Methods**			
Study design	4	Present key elements of study design early in the paper	2
Setting	5	Describe the setting, locations, and relevant dates, including periods of recruitment, exposure, follow-up, and data collection	2
Participants	6	(*a*) Give the eligibility criteria, and the sources and methods of selection of participants. Describe methods of follow-up	2–3
		(*b*) For matched studies, give matching criteria and number of exposed and unexposed	N/A
Variables	7	Clearly define all outcomes, exposures, predictors, potential confounders, and effect modifiers. Give diagnostic criteria, if applicable	3–4
Data sources/ measurement	8	For each variable of interest, give sources of data and details of methods of assessment (measurement). Describe comparability of assessment methods if there is more than one group	3–4
Bias	9	Describe any efforts to address potential sources of bias	4–5
Study size	10	Explain how the study size was arrived at	2–3*
Quantitative variables	11	Explain how quantitative variables were handled in the analyses. If applicable, describe which groupings were chosen and why	4–5
Statistical methods	12	(*a*) Describe all statistical methods, including those used to control for confounding	4–5
		(*b*) Describe any methods used to examine subgroups and interactions	5
		(*c*) Explain how missing data were addressed	N/A
		(*d*) If applicable, explain how loss to follow-up was addressed	3–4
		(*e*) Describe any sensitivity analyses	5
**Results**			
Participants	13	(a) Report numbers of individuals at each stage of study—e.g. numbers potentially eligible, examined for eligibility, confirmed eligible, included in the study, completing follow-up, and analyzed	3, 5–6
		(b) Give reasons for non-participation at each stage	3, 6
		(c) Consider use of a flow diagram	N/A†
Descriptive data	14	(a) Give characteristics of study participants (e.g. demographic, clinical, social) and information on exposures and potential confounders	5–7
		(b) Indicate number of participants with missing data for each variable of interest	N/A
		(c) Summarize follow-up time (e.g., average and total amount)	3
Outcome data	15	Report numbers of outcome events or summary measures over time	6–8

*No formal power analysis was performed due to the population-based design of the study.
†Specific numbers illustrating the sample sizes included in each stage of the analyses are embedded in the main text of the paper (pages 2–3).

Table S2. Description of Data Sources.

Data source	Description
Hospital Discharge Records	In operation since 1994, the database includes demographic characteristics (age, sex and health district), admission and discharge dates, primary diagnosis, up to five secondary diagnoses, up to 11 procedures, and discharge status for each patient discharged from either public or accredited private hospitals. Diagnoses and procedures are categorized using the ICD-9-CM, version 2007. The records are submitted by all hospitals operating in Emilia-Romagna to the Regional Authority and, following data quality control, regularly sent from the Regional Authority to the Ministry of Health.
Residential Care Discharge Records	Operational since 2008, it comprises obligatory data on patients discharged from non-profit or accredited private facilities for mental health-related hospital services, including admission and discharge dates, primary diagnosis, and discharge status. Accredited facilities refer to private hospitals where fees are reimbursed by the Italian National Health Service if the patient is an official resident in Italy.
Mental Health Information System	In operation since 2005 for administrative and clinical epidemiological purposes. The database records all adult patients who have at least one contact with the Community Mental Health Centers (CMHCs), including their demographic characteristics, ICD-9-CM diagnoses, and details of each type of intervention administered.
Outpatient and directly dispensed Pharmaceutical Database	Operational since 2002, the database pertains to drugs reimbursed by the healthcare system that are prescribed by the family doctor or a specialist (AFT), or directly dispensed by hospital pharmacies (FED). It includes information on patients' demographics (age, sex and health district), prescriptions (substance name, ATC System code—V.2013, trade name, date of prescription, date of dispensation, and number of packages), as well as prescribers.
Specialistic Ambulatory Care	In operation since 2002, the database includes outpatient visits, instrumental examinations, and laboratory tests provided to individual patients by public and accredited private providers in Emilia-Romagna. It collects information about medical services, providers, patients, and prescribing doctors. Mental health services are not included in the database.
Vital Registration System	Operational since 1995, it includes information on patients' date, place, and cause of death classified according to the ICD-10.

Abbreviations: ICD-9-CM, International Classification of Diseases, 9th Revision, Clinical Modification; ICD-10, International Classification of Diseases, 10th Revision.

Table S3. ICD-9-CM Diagnosis Codes Used to Identify Depression

Description	Code
Depression	
Major depressive disorder single episode	2962
Major depressive disorder recurrent episode	2963
Other and unspecified affective psychoses	2969
Dysthymic disorder	3004
Adjustment disorder with depressed mood	3090
Adjustment reaction with prolonged depressive reaction	3091
Depressive disorder not elsewhere classified	311

*ICD-9-CM procedure codes.
Abbreviations: ICD-9-CM, International Classification of Diseases, 9th Revision, Clinical Modification.

Table S4. Adjusted Incidence Rate Ratios of Recommended Annual Diabetes Exams (GCI) Associated with One-Year Increases in Duration of Post-Diabetes Depression, Overall and by Age Group ad Diabetes Onset.

Year	All			Adults (<65 y)			Older Adults (≥65 y)		
	IRR	95%CI	*P*-value	IRR	95%CI	*P*-value	IRR	95%CI	*P*-value
2018	0.95	0.80, 1.14	0.579	0.95	0.71, 1.28	0.746	0.98	0.78, 1.23	0.838
2019	0.94	0.84, 1.06	0.310	1.01	0.85, 1.20	0.913	0.89	0.77, 1.03	0.129
2020	0.98	0.89, 1.08	0.719	0.99	0.86, 1.15	0.921	0.96	0.84, 1.09	0.491
2021	0.97	0.90, 1.05	0.496	0.99	0.89, 1.11	0.892	0.96	0.87, 1.07	0.454
2022	0.99	0.94, 1.05	0.855	0.98	0.93, 1.09	0.840	0.98	0.90, 1.06	0.593

Notes: IRRs were obtained via covariate adjustment. Depression duration varies by construction according to the year being analyzed: up to three years for 2018, up to four years for 2019, up to five years for 2020, up to six years for 2021, and up to seven years for 2022.
Abbreviations: GCI, Guideline Composite Indicator; IRR, Incidence Rate Ratio; CI, Confidence Interval.
